# Telemedicine in the clinical care of Chagas disease and American cutaneous leishmaniasis: pilot study in a public referral hospital in Brazil

**DOI:** 10.3389/fpubh.2025.1616368

**Published:** 2025-06-26

**Authors:** Fernanda Gonçalves Ferreira Salvador, Liliane Fátima A. Oliveira, Maria Inês Fernandes Pimentel, Marcelo Rosandiski Lyra, Alejandro Marcel Hasslocher-Moreno, Marcelo Teixeira Holanda, Margareth Catoia Varela, Henrique Silveira, Cláudia Maria Valete

**Affiliations:** ^1^Global Health and Tropical Medicine (GHTM), Associate Laboratory in Translation and Innovation Towards Global Health (LA-REAL), Institute of Hygiene and Tropical Medicine (IHMT), NOVA University Lisbon (UNL), Lisbon, Portugal; ^2^Clinical Research and Surveillance Laboratory in Leishmaniasis, Evandro Chagas National Institute of Infectious Diseases (INI) Oswaldo Cruz Foundation (Fiocruz), Rio de Janeiro, Brazil; ^3^Clinical Research Laboratory in Chagas Disease, Evandro Chagas National Institute of Infectious Diseases (INI) Oswaldo Cruz Foundation (Fiocruz), Rio de Janeiro, Brazil; ^4^Health Surveillance and Immunization Research Unit (LIVS), Evandro Chagas National Institute of Infectious Diseases (INI) Oswaldo Cruz Foundation (Fiocruz), Rio de Janeiro, Brazil; ^5^Department of Otorhinolaryngology and Ophthalmology, School of Medicine, Federal University of Rio de Janeiro, Rio de Janeiro, Brazil

**Keywords:** Chagas disease, cutaneous leishmaniasis, digital health, neglected tropical diseases, telemedicine

## Abstract

**Background:**

Neglected tropical diseases (NTDs) remain major public health challenge in low- and middle-income countries (LMICs), especially in rural and underserved regions. Although telemedicine has expanded globally, evidence on its implementation for individual clinical care of NTDs remains scarce. This pilot implementation study aimed to assess the feasibility and acceptability of synchronous telemedicine consultations for patients with Chagas disease (CD) and American Cutaneous Leishmaniasis (ACL) in a national reference hospital in Brazil.

**Methods:**

We conducted a mixed-methods implementation study using the Design Science Research Methodology (DSRM). The context analysis involved structured surveys with 31 health professionals and semi-structured interviews with two local managers. Patients diagnosed with CD or ACL were recruited from August to October 2024. Teleconsultations were performed via WhatsApp video calls by four specialists. Patient satisfaction was evaluated using structured questionnaires.

**Findings:**

Of the 46 patients recruited, 38 (82.6%) completed teleconsultations. Most were older adults and had no prior telehealth experience. Despite low digital literacy and education levels, 63.2% (24/38) completed the teleconsultation without assistance. High levels of satisfaction were reported: 97.4% (37/38) were satisfied and would attend future virtual visits. Participants reported the reduced travel burden and improved access to care. No major technical issues were observed.

**Conclusion:**

Synchronous telemedicine via a low-cost, widely available platform was feasible and well-accepted for clinical follow-up of CD and ACL in a Brazilian public hospital. This approach may help reduce access barriers to specialized care for NTDs. Further research should evaluate clinical outcomes and cost-effectiveness in LMIC settings.

## 1 Introduction

Neglected Tropical Diseases (NTDs) comprise 21 diseases recognized by the World Health Organization (WHO) that affect around 1 billion people globally (1). In 2021, 199 countries reported at least one NTD, making their control a challenge to the Sustainable Development Goals (SDGs) 2030 (2). These diseases are prevalent in poor and vulnerable regions where access to health care is limited and can lead to disability, stigma, and lasting socioeconomic impact, even after a cure. Universal health coverage is an essential pillar for their control (3, 4).

Many NTDs are endemic in rural and remote areas with limited healthcare support networks. In this scenario, digital health innovations have emerged as a promising strategy for expanding access to healthcare through remote diagnostic technologies, epidemiological surveillance, and virtual clinical follow-up (5–7). Telemedicine can be defined by WHO as “the delivery of health care services, where distance is a critical factor, by all healthcare professionals using information and communication technologies for the purposes of diagnosis, treatment and prevention of diseases and injuries” (6). Despite the expansion of this resource after the Covid-19 pandemic (8), low- and middle-income countries (LMICs) face barriers to its implementation, especially regarding access to internet connection. In Brazil, general connectivity data indicate that, on average, 84% of the population uses the internet (9). However, access is much lower in rural areas (10), exposing the country’s immense geographic inequalities. For example, in the northern region, which has the highest NTD detection rate in Brazil (11), some municipalities have internet coverage below 10%, comparable to levels in low-income African countries (9, 10).

Regarding the effectiveness of telemedicine for clinical care, evidence comparing in-person consultations with synchronous remote ones shows that it is effective in increasing the early detection of diseases and complications, including infectious diseases (12, 13). In Brazil, video consultations have been shown to reduce the need for in-person medical evaluations for patients with mild to moderate dengue, and they have also demonstrated reliable diagnostic accuracy in the assessment of leprosy (14, 15). Despite occasional experiences identified for NTDs, robust evidence on the implementation of telemedicine in public services for individual clinical care remains scarce in the literature (15–17).

Brazil has shown great progress in investments in Digital Health for the Unified Health System (Sistema Único de Saúde, SUS), being an area of expansion in the government and in the private sector (16–18). Legal authorization and certainty for the practice of medical remote care by telemedicine are recent in the country, established by Law 14,510/2022 (19, 20). On the other hand, the scarcity of specific protocols to direct care in remote mode and the slow incorporation of digital skills into teaching in medical schools are still important limitations to be addressed (18).

Brazil has 2.18 doctors per 1,000 inhabitants, below high-income American countries such as Canada and the United States, with 2.7/1000 each (21). The country’s territorial extension and unequal geographic distribution of professionals make the difficulty of accessing medical consultations a major public health problem. A 2018 survey showed that focal specialists are concentrated mainly in the South and Southeast regions, to the detriment of regions where the main areas of occurrence of NTDs are found (11, 21). For example, 57.8% of the country’s infectious disease specialists work in the southeast region, while the north region has only 6.3% of these specialists (21). The use of telemedicine for remote care of diseases that require greater complexity could be useful in ensuring equal access to qualified health care.

Leishmaniasis and Chagas disease remain NTDs with a relevant impact on public health in Brazil (22), and their effective control requires integrated surveillance models and intersectoral collaboration, aligned with a One Health approach (23). American Cutaneous Leishmaniasis (ACL) is endemic in 19 countries in the Americas (24), and in all five regions of Brazil, with a higher prevalence in the North, Central-West, and Northeast regions. It is caused by protozoa of the genus *Leishmania* transmitted by phlebotomine insects of the genus *Lutzomyia*. Transmission occurs through the bite of the infected female of the insect vector. Cutaneous Leishmaniasis usually presents with single or multiple ulcerated lesions, mainly in areas not covered by clothing. Mucosal Leishmaniasis can affect the nose, mouth, pharynx, and/or larynx, requiring early diagnosis and immediate treatment, as it is potentially disfiguring and debilitating. Specific treatment for ACL, offered free of charge by SUS in Brazil, may involve oral, intralesional, intramuscular, or intravenous medications (25).

Chagas disease (CD) is endemic in 21 countries in the Americas (26). Its main transmission mechanism is insect vectors of the Triatominae subfamily, and other main routes include vertical/congenital, oral through food contaminated by *Trypanosoma cruzi*, transfusion, or organ transplants. The disease has acute and chronic clinical phases, the latter with three well-defined clinical forms: indeterminate (60% of cases), cardiac (30%), and digestive (10%) (27). Patients with CD may develop cardiac alterations with a high impact on morbidity and mortality (27), and etiological treatment is provided by the SUS (28). CD and ACL still face significant barriers in relation to diagnosis, with an impact on underreporting and public policy formulation. In addition, addressing the main social determinants of health, such as challenges related to migration, is essential to expanding access to comprehensive care of them (29).

To the authors’ knowledge, this is the first telemedicine pilot study to demonstrate the results of synchronous care between doctors and patients for these two diseases in a public service in Brazil. This study aimed to evaluate the feasibility and acceptability of telemedicine as a care strategy for NTDs in a SUS infectious disease referral hospital in the city of Rio de Janeiro. This objective is in line with Pan American Health Organization’s recommendations for expanding access to digital health in the Americas region (30).

## 2 Materials and methods

This is an implementation study based on mixed methods that made it possible to identify and explore the opportunity of using digital health solutions in a specific context of a tertiary-level outpatient health service (31–34). Using the Design Science Research Methodology (DSRM) developed by Peffers (35), a pilot implementation of telemedicine for the care of NTDs was designed, demonstrated, and evaluated in this specific setting.

### 2.1 Study design

**Figure 1:**
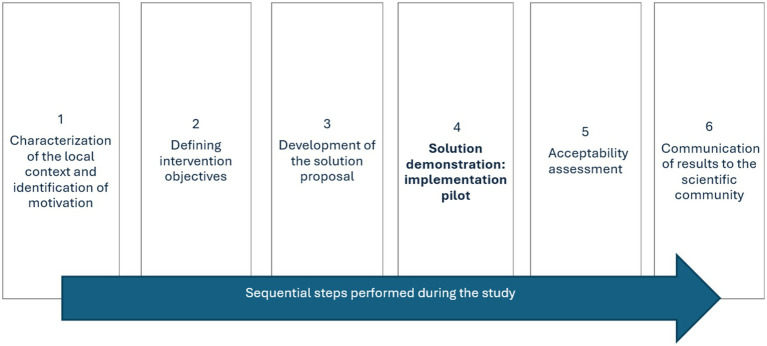
DSRM applied to research.

According to DSRM (35), the study structure was based on a process with 6 sequential steps: 1. characterization of the context and identification of the motivation of the actors involved in solving the problem; 2. definition of objectives; 3. design and development of an intervention to solve a problem found in the context; 4. demonstration of the solution; 5. evaluation of acceptability; and 6. communication of results, detailed in Figure 1.

In stage 1, quantitative methodologies (structured questionnaires with health professionals) and qualitative (semi-structured interviews with local managers) were used. The analysis of the local profile and the suggestions arising from this stage contributed to the design and development of the telemedicine utilization model applied in stages 2, 3, and 4. In stage 5, quantitative methodologies were used (structured questionnaires with patients who underwent teleconsultation at the service). Table 1 presents the methodological details of each of these stages.

**Table 1:** Design of the mixed methods study performed.

Objective	Methodology
Context and motivation evaluation—health professionals	Quantitative: structured questionnaires with health professionals working in the clinical care of NTDs at the service (*n* = 31)
Context evaluation and motivation—local managers	Qualitative: interviews using semi-structured scripts with local managers and content analysis (*n* = 2)
Context evaluation—patients	Quantitative: structured questionnaires with ACL and CD patients recruited for the research (*n* = 46)
Feasibility evaluation—pilot demonstration	Proof of concept: intervention using teleconsultations for ACL and CD with doctors and patients of the service
Acceptability evaluation—patients	Quantitative: structured questionnaires with ACL and CD patients after telemedicine care (*n* = 38)

ACL, American Cutaneous Leishmaniasis; CD, Chagas Disease.

### 2.2 Ethical aspects

This study was approved by the Ethics Committee of the Evandro Chagas National Institute of Infectology of the Oswaldo Cruz Foundation (INI-Fiocruz) approval No. 6.933.545 CAAE: 59059622.9.0000.5262 dated 06/08/2022. All participants signed an informed consent form which was read aloud and thoroughly explained to those with low or no literacy to ensure full understanding before consent was obtained. All requirements of the general data protection law in force in the country were followed (36). Patient medical interactions were conducted in accordance with the Brazilian Medical Code of Ethics.

### 2.3 The study scenario

INI-Fiocruz provides teaching, research, and clinical care in infectious diseases, including outpatient consultations, hospital admissions, and day hospitalizations; laboratory and imaging diagnostic tests; and the study of infectious diseases through scientific and technological research projects. The service was chosen because it presents important structural and conjunctural conditions for carrying out this study: clinical staff with expertise in NTDs, internet access in all offices, high daily volume of patients with a confirmed diagnosis of NTDs, and local managers interested in promoting innovative changes. Among the Institute’s laboratories are the Clinical Research and Surveillance Laboratory for Leishmaniasis and the Clinical Research Laboratory for Chagas disease (37), which include specific outpatient clinics organized according to disease specialty that constitute national references in the SUS.

Context analysis is a crucial factor for implementation research and encompasses the first two stages of the DSR methodology (35). For this initial exploratory phase, two in-person interviews were conducted with local service managers selected due to their strategic roles. The data was subjected to a manual categorical content analysis. After automated transcription using the Sonix platform, a coding map was developed, from which the main analytical categories emerged through an inductive process, as proposed by Bardin (38). Within this framework, thematic analysis was applied to identify key patterns and meanings. Subthemes were then systematically categorized to further organize and deepen the understanding of the identified topics. This process aimed to map motivations and managerial recommendations to the development of the pilot. The interviewer received adequate training to ensure methodological rigor and reliability throughout the process. Additionally, detailed field notes were collected to complement the transcripts and enrich the analysis.

Health professionals were also evaluated through structured questionnaires containing 40 questions. The instrument was sent via an anonymized link by email to 65 participants of the service, with the following inclusion criteria: being over 17 years old, being a higher education professional with a permanent link to the service and providing care in multidisciplinary teams of NTDs care. All eligible professionals were recruited. The study data were collected and managed using free electronic REDCap data capture tools hosted on [Fiocruz] (39). The instruments are presented in Appendices I, II. To ensure content validity, the questionnaire was pre-tested with five users (excluded from the final analysis) to assess clarity and relevance. The REDCap platform’s pre-production mode was used to verify functionality, data accuracy, and anonymization.

### 2.4 Study team

Four medical professionals volunteered to participate in the project: two dermatologists specialists in ACL, an infectious disease specialist in CD, and a cardiologist specialist in CD. The project also had the local support of a nurse specialist in tropical infectious diseases.

### 2.5 Choosing WhatsApp

Low digital literacy and low educational level of patients are a central concern for the implementation of telemedicine in LMIC, including populations affected by NTDs (6, 7, 15, 30). WhatsApp was chosen as the communication channel for teleconsultation, appointments, and sending the evaluation questionnaire due to its popularity and ease of use (40). This feature is an instant messaging, voice and video calling application that allows sharing of photos and documents via mobile phone or desktop version (WhatsApp-Web). Its use as a potential telecommunications tool in medicine has been explored more recently (41).

Strict measures were implemented to safeguard data security and privacy. The use of WhatsApp was strictly confined to research-related communication, as explicitly authorized in the Informed Consent Form (ICF) (Appendix V), which details the primary measures adopted. All procedures fully complied with the Brazilian General Data Protection Law and the Telehealth Brazilian Laws (19, 20, 36). Teleconsultations were conducted using password-protected devices and secure internet connections. Participant data were securely stored on an institutional platform featuring encryption, restricted access, and two-factor authentication, accessible solely to the research team. Although institutional telemedicine protocols were not yet formally established at the time at the hospital, all processes adhered to applicable legal and ethical standards, and the intervention was deliberately designed to address this structural gap in healthcare delivery.

### 2.6 Inclusion criteria, recruitment, and identification of patient profiles

Patient recruitment was carried out in person between August 14 and October 16, 2024. Inclusion criteria were: having a confirmed diagnosis of CD or ACL, being over 17 years old, having a fixed or mobile device with internet access and WhatsApp installed at home or at work, being interested in having a follow-up appointment with their referring physician via telemedicine, and not having any health condition for which the responsible physician preferred an in-person follow-up appointment. All patients who met these criteria and who attended an in-person consultation during this period with one of the four participating physicians were approached, constituting a convenience sampling method. Patients with different clinical stages of CD and different evolutionary moments of ACL were recruited. Appointments were limited by the usual availability of service vacancies.

Upon accepting to participate, patients completed a 12-question questionnaire in person on the team’s tablet (Appendix III). Data were collected and managed in REDCap (37). Each teleconsultation appointment was confirmed twice by WhatsApp message (Appendix IV; Supplementary Figure S1). Patients who reported low education level or low digital literacy were advised to ask for help from a family member or friend.

### 2.7 Carrying out teleconsultations: demonstrating the solution

**Figure 2:**
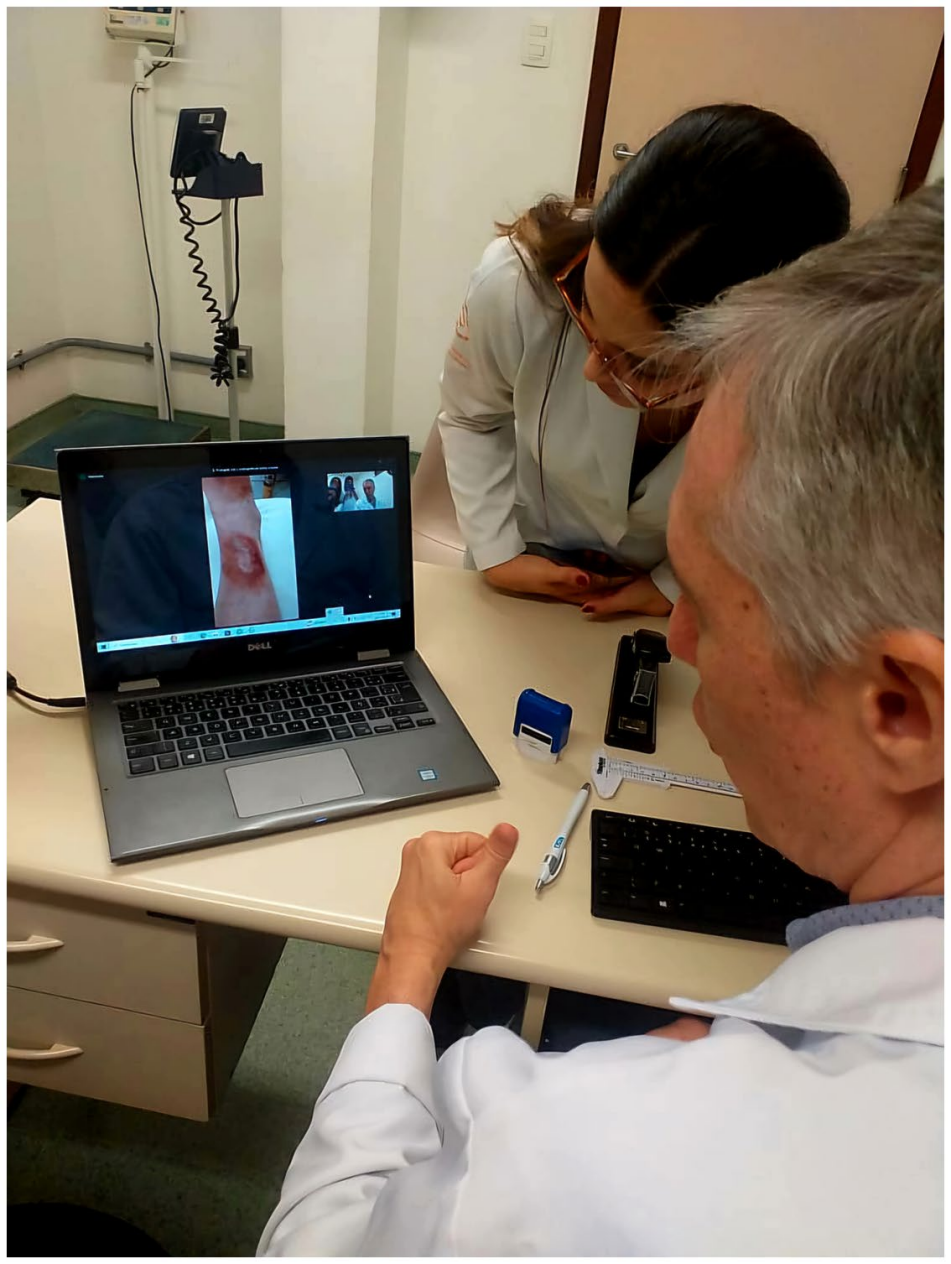
Specialist physician conducting a remote consultation for Leishmaniasis follow-up during the study. Credits: The author. The individual depicted provided written consent for the publication of this image. License: CC-BY.

The intervals between in-person consultations and the clinical follow-up teleconsultation ranged from 1 to 7 weeks (September 18–November 6, 2024). Simple video calls from a project-exclusive WhatsApp account were used, made from a portable notebook using the hospital’s institutional WiFi network with approximately 30 Mbps (Figure 2; Supplementary Figures S2–S4). The images were captured by the notebook’s camera. Healthcare professionals may opt to use headsets or not.

### 2.8 Patient evaluation after teleconsultation: user satisfaction

The evaluation of the solution corresponds to the penultimate stage of the DSRM. Immediately after the end of the teleconsultation, a link to a structured evaluation questionnaire with six questions prepared by the team based on the adapted version of a scale validated in Brazil for this purpose was sent via WhatsApp instant message, covering the following areas: general satisfaction, convenience, and communication skills (Appendix III) (42). Although the use of immediately post-consultation questionnaires may introduce response bias to social desirability or gratitude toward providers, this risk was minimized by distributing then through a channel managed exclusively by the research team, with no access granted to the physicians. Participants completed them at their convenience, outside the consultation context.

### 2.9 Statistical analysis

Frequencies were calculated for categorical variables and measures of central tendency (median, minimum and maximum) for continuous variables. The comparison between the patients who needed help to do the teleconsultations and those who did not was made using the chi-square test for category variables and the Mann–Whitney test for continuous variables. The analyses were carried out using statistical software Statistical Package for Social Sciences version 19.0 (IBM Corp, Armonk, NY, United States). The confidence interval used was 95% with a significant level of 5% (*p* < 0.05).

## 3 Results

### 3.1 Characteristics of the context, motivation and objectives of the solution

#### 3.1.1 Healthcare managers

The interviews with healthcare managers provided valuable insights into the local history and previous experiences with remote service, and helped assess interest in the pilot while gathering suggestions for its development. The categorical analysis identified converging factors in the participants’ statements, which were organized into five recurring thematic categories and 20 subcategories, is presented in Supplementary Box S1.

The main common points among the managers are the perception of a local scenario that is receptive to the incorporation of digital health and the desire for the service to have its own telehealth platform in the future. The following main concerns also stand out: patient safety, ethical and legal aspects related to confidentiality and protection of sensitive data, compliance with the workload of professionals in the event of hybrid working hours, and adequate ambience and equipment, including acoustics and informatic support. Regarding patient users’ expectations of adherence, low levels of education and low income for internet access emerged as the main barriers.

#### 3.1.2 Health professionals

Of the 65 questionnaires sent to health professionals, 31 (47.7%–31/65) were completed in full. The median age of the participants was 48, 54.8% were doctors, and the rest were other health professionals. The median length of service was 16 years. The NTDs most attended by the participants were CD (48.4%), ACL (38.7%) and dengue fever/Chikungunya (32.3%), as well as systemic and implant mycoses, schistosomiasis, leprosy, snakebite poisoning, rabies prophylaxis and others. The profile of the health professionals and the main results from the initial stage of context analysis are shown below in Table 2.

**Table 2:** Profile of health professionals.

Gender at birth	n (%)
Female	20 (64.5)
Male	11 (35.5)
Age in years	Median (min–max)
Age	48 (30–63)
Professional category	n (%)
Medical specialist	16 (51.6)
Nurse	4 (12.9)
Pharmaceutical	3 (9.7)
Physiotherapist	2 (6.5)
Veterinarian	2 (6.5)
Nutritionist	1 (3.2)
Public health technician	1 (3.2)
Speech therapist	1 (3.2)
General practitioner	1 (3.2)
Working time in years at INI	Median (min–max)
Working time	16 (3–35)
Diseases you work with^a^	n (%)
Chagas	15 (48.4)
Leishmaniasis	12 (38.7)
Chikungunya	10 (32.3)
Dengue	10 (32.3)
Mycetoma, chromoblastomycosis and other deep mycoses	9 (29.0)
Scabies and other ectoparasitoses	5 (16.1)
Snakebite poisoning	5 (16.1)
Leprosy	4 (12.9)
Geohelminthiasis	3 (9.7)
Schistosomiasis	2 (6.5)
Onchocerciasis	1 (3.2)
Rabies	1 (3.2)
Lymphatic Filariasis	1 (3.2)
Teniasis and Cysticercosis	1 (3.2)
Toxoplasmosis	1 (3.2)
Others	2 (6.5)

^a^n, absolute number; a, some professionals worked with more than one disease.

The interest in telemedicine and the main facilitators and challenges highlighted by INI health professionals for the implementation of a pilot applied to NTDs are presented in Table 3. The database for this stage, with identifying data hidden, is available at Supplementary database S1.

**Table 3:** Interest in telemedicine and key facilitators and challenges identified by health professionals for implementing a pilot project focused on NTDs.

How comfortable do you feel conducting teleconsultations with patients via video calls?	n (%)
Enough/Sufficient	14 (45.2)
Little/Very little	12 (38.7)
Indifferent	5 (16.1)
How comfortable are you discussing clinical cases with professionals from other healthcare services via video calls?	n (%)
Enough/Sufficient	17 (54.8)
Little/Very little	9 (29.0)
Indifferent	5 (16.1)
What are the main reasons for not using remote communication more frequently for clinical case discussions?	n (%)
Lack of availability in the service	13 (41.9)
I don’t see the need/benefits	5 (16.1)
Lack of technical support	4 (12.9)
No time	3 (9.7)
Current use is deemed sufficient	1 (3.2)
Other reason	5 (16.1)
Do you believe the implementation of telemedicine can improve care for NTDs in your service?	n (%)
Yes (a little/ a lot)	27 (87.1)
No	4 (12.9)
Would you be willing to participate in a pilot project of a telemedicine platform focused on NTD care in this service?	n (%)
Yes	15 (48.4)
Maybe	12 (38.7)
No	4 (12.9)
In your opinion, what are the main advantages of using video calls for telemedicine care of patients with NTDs in this service? (Select up to 3 options)	n (%)
Improved access and continuity of care, reduced absenteeism	16 (51.6)
Improved treatment adherence monitoring	15 (48.4)
Monitoring of critical clinical events and complications	14 (45.2)
Support for epidemiological surveillance and contact tracing	11 (35.5)
Contribution to clinical research data collection	11 (35.5)
Access to specialists from other services or regions for clinical case discussions	6 (19.4)
Support during outbreaks in remote areas	6 (19.4)
Improved coordination of community-based health initiatives in the local areas	2 (6.5)
Optimized scheduling and management of consultation time	2 (6.5)
And what are the main challenges to telemedicine care of patients with NTDs in this service? (Select up to 3)	n (%)
Low patient education and digital literacy	18 (58.1)
Operational difficulties in accessing the internet and network connections in this service	17 (54.8)
Limited access to technical support	14 (45.2)
Lack of appropriate technological equipment	12 (38.7)
Potential workloads increase and workflow disruption	9 (29.0)
Data security and confidentiality concerns	7 (22.6)
Reduced consultation quality due to virtual format	7 (22.6)
Limited digital proficiency of the team	2 (6.5)
Lack of knowledge about ethical/legal frameworks	1 (3.2)

n, absolute number.

### 3.2 The pilot study

**Figure 3:**
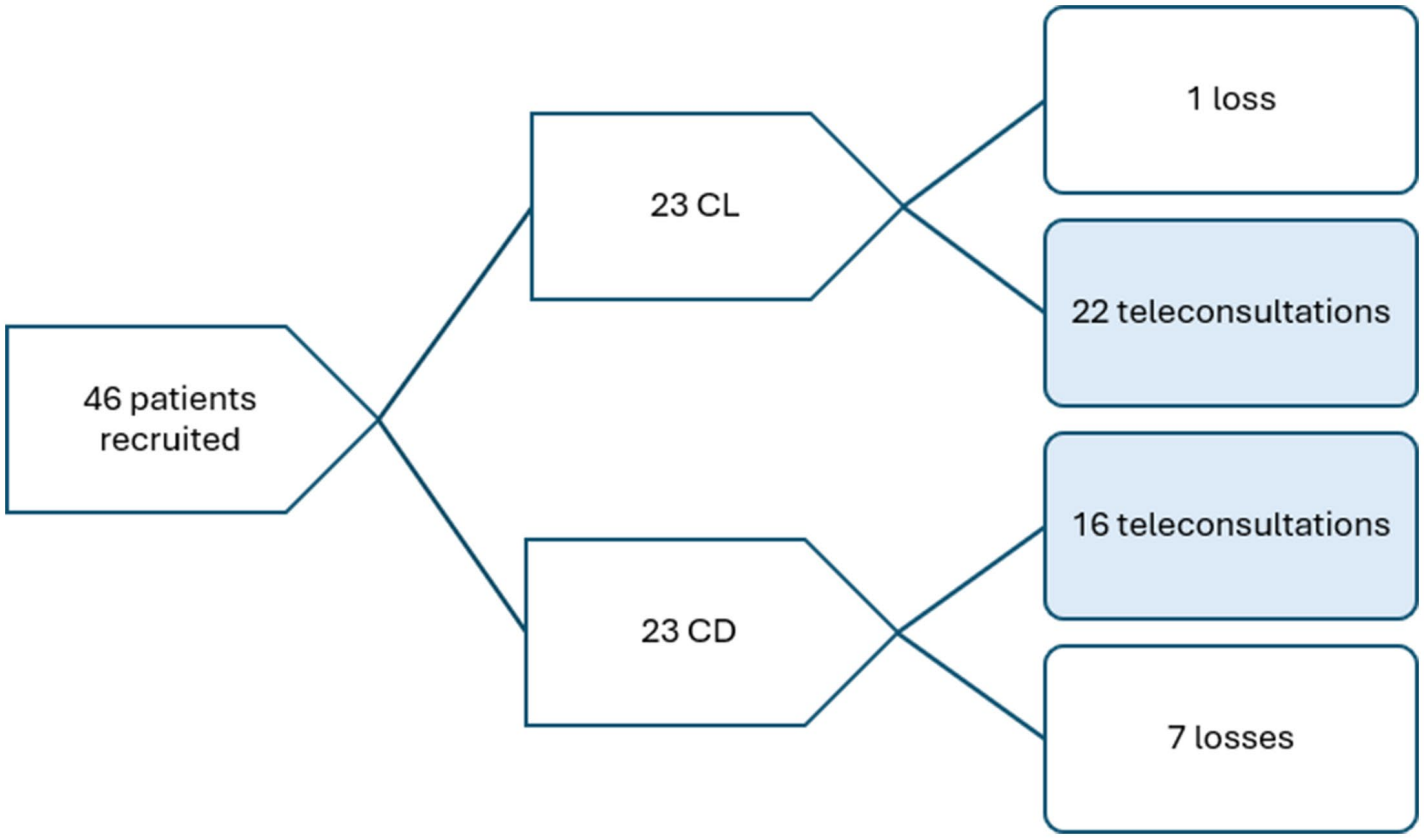
Utilization flowchart.

Based on the hospital’s electronic health records, 29 patients with ACL and 72 with CD, identified by ICD-10 codes, were seen by the four physicians during the work shifts in which recruitment was conducted. Of these, 46 (45.5%) met the eligibility criteria and consented to participate, resulting in 38 completed teleconsultations. The utilization flowchart can be seen in Figure 3.

#### 3.2.1 ACL group characteristics

Of the 23 patients with ACL recruited, 22 received care via telemedicine. Only one patient canceled due to a power outage in his city on the scheduled day. Of these 23, 18 patients had a history of solely cutaneous presentation, three patients had mucosal lesions, and two patients were classified as having disseminated Leishmaniasis. Regarding the stage of the disease on the day of recruitment, 5 new cases were detected on the day of diagnosis, 14 were already being monitored for cure control, three were treated for recurrence of lesions and one for treatment failure associated with AIDS.

#### 3.2.2 Characteristics of the CD group

Twenty-three patients with CD were recruited, six had advanced Chagas heart disease and 17 had the indeterminate clinical form of the disease. Of the total of 23 patients with CD recruited, 16 received care via telemedicine. Among patients with indeterminate presentation, four (24%) did not undergo teleconsultation: one due to hearing impairment; two (from the same family) due to scheduling conflicts with other medical examinations on the same date; and one canceled the appointment. Among patients with heart disease, three (50%) did not perform the teleconsultation: one changed the modality to in-person by personal choice, one (illiterate) had difficulties communicating with the team and missed the appointment date, and one canceled.

#### 3.2.3 General technical aspects of teleconsultations

Regarding the hospital’s internet connection, which had previously been a concern for managers and healthcare professionals, no teleconsultations were canceled for this reason, although there were occasional interruptions that were quickly resolved, without compromising the duration or achievement of the teleconsultation objectives. The sound and audio quality and image resolution were generally sufficient.

Sending electronic prescriptions and exam requests was necessary for most appointments. Since the hospital did not provide certified digital signatures, the documents were sent in two ways: they were generated free of charge by the Rio de Janeiro State Regional Medical Council’s website, with the professional’s private digital signature (43); or the documents were printed, signed, stamped, and scanned. In both cases, the documents were sent to patients via WhatsApp.

#### 3.2.4 Patient profile and satisfaction with teleconsultations

Around 57% of the patients were aged 60 or over at the time of recruitment. The majority had a low level of education, lived in another municipality, and had no previous experience with remote medical teleconsultations, with the pilot teleconsultation being their first experience of remote contact with a health professional. Despite this, the majority said they had already made video calls and said they had not needed help from a family member or friend to make the teleconsultations. Tables 4, 5 present a summary of the profile of the patients seen and the assessments carried out by the patients after the teleconsultation.

**Table 4:** Sociodemographic profile of patients recruited for the pilot study.

Variable	Leishmaniasis (*n* = 23)	Chagas (*n* = 23)
Gender assigned at birth	n (%)	n (%)
Male	11 (47.8)	11 (47.8)
Female	12 (52.2)	12 (52.2)
Municipality	n (%)	n (%)
Rio de Janeiro	5 (21.7)	11 (47.8)
Other	18 (78.3)	12 (52.2)
Illiterate	n (%)	n (%)
Yes	5 (21.7)	6 (26.1)
No	18 (78.3)	17 (73.9)
Up to the 5th year of elementary school	n (%)	n (%)
Yes	9 (39.1)	13 (56.5)
No	14 (60.9)	10 (43.5)
Age	Median (minimum-maximum)	Median (minimum-maximum)
Paticipants age	65 (18–79)	63 (43–91)
Do you have access to a smartphone, tablet or computer with internet access?	n (%)	n (%)
Yes	22 (95.7)	23 (100)
No	1 (4.3)	0 (0)
Have you ever talked to another person via long-distance video calls (on platforms such as WhatsApp, zoom, skype, google meet etc.)? n (%)	n (%)	n (%)
Yes	19 (82.6)	21 (91.3)
No	4 (17.4)	2 (8.7)
Did you find it easy to talk to another person via long-distance video calls?^a^ n (%)	n (%)	n (%)
Yes	15 (78.9)	16 (69.6)
No	4 (21.1)	5 (21.7)
Have you ever made a medical appointment via video call? n (%)	n (%)	n (%)
Yes	4 (17.4)	0 (0)
No	19 (82.4)	23 (100)
Did you enjoy making medical appointments by video call?^b^ n (%)	n (%)	n (%)
Yes	3 (75.0)	
Indifferent	1 (25.0)	
Do you think you will need help from someone else to be able to do this medical teleconsultation via video call?	n (%)	n (%)
Yes	13 (56.5)	11 (47.8)
No	10 (43.5)	12 (52.2)

a n referring to the 19 CL patients and 21 CD patients who had already made video calls.

b n referring to the 4 CL patients who had already undergone teleconsultations.

n, absolute number.

**Table 5:** Evaluation of patient satisfaction with teleconsultations.

Variable	Leishmaniasis (*n* = 22)	Chagas (*n* = 16)
Are you satisfied with being attended remotely via telemedicine by your INI-Fiocruz healthcare team?	n (%)	n (%)
Yes	22 (100)	15 (93.8)
Indifferent		1 (6.2)
Were you able to explain your problems clearly to the healthcare professional during telemedicine care?	n (%)	n (%)
Yes	22 (100)	14 (87.5)
Not applicable		2 (12.5)
Do you need help from someone else to complete this teleconsultation?	n (%)	n (%)
Yes	9 (40.9)	5 (31.2)
No	13 (59.1)	11 (68.8)
Has telemedicine saved you time and/or money on travel?	n (%)	n (%)
Yes	22 (100)	15 (93.8)
No		1 (6.2)
Would you set up a new telemedicine service with your INI-Fiocruz team?	n (%)	n (%)
Yes	22 (100)	15 (93.8)
Indifferent		1 (6.2)
Would you recommend telemedicine care to other patients?	n (%)	n (%)
Yes	22 (100)	15 (93.8)
Indifferent		1 (6.2)

n, absolute number.

Among the most frequent cities of origin for patients outside the capital, most are characterized by either substantial rural zones (e.g., São José do Vale do Rio Preto), mountainous areas with agricultural activity (e.g., Teresópolis, Miguel Pereira), or peripheral communities with limited access to healthcare (e.g., Magé, Itaboraí).

Most of the patients who received the consultation expressed excellent overall satisfaction with the teleconsultation, having been able to explain their health problems clearly and expressed an interest in having another remote consultation with the team. When we compared the group that needed help with carrying out the teleconsultation to those who did not, it was observed that in ALC, there were significant differences regarding the level of education and age. All the illiterate patients in the ALC group needed help to make the teleconsultation and the median age among those who needed help was also higher. This difference was not observed in the CD group Table 6. Databases excluding patient identifiers are full available at Supplementary databases S2, S3.

**Table 6:** Age and education level according to the need for support from others in carrying out the video teleconsultation.

Variable		Reported need for support							
		Leishmaniasis				Chagas			
		Total (*n* = 22)	Yes (*n* = 9)	No (*n* = 13)	*p*-value	Total (*n* = 16)	Yes (*n* = 5)	No (*n* = 11)	*p*-value
**Illiterate n (%)**	Yes	4 (18.2)	4 (44.4)	0 (0.0)	0.017*	2 (12.5)	2 (40.0)	0 (0.0)	0.083
	No	18 (81.8)	5 (55.6)	13 (100)		14 (87.5)	3 (60.0)	11 (100)	
**Up to the 5th (%)**	Yes	8 (36.7)	7 (77.8)	1 (7.7)	0.001*	8 (50.0)	3 (60.0)	5 (45.5)	1
	No	14 (63.3)	2 (22.2)	12 (92.3)		8 (50.0)	2 (40.0)	6 (54.5)	
**Median age in years (min–max)**		63 (18**–**77)	70 (41**–**77)	45 (18**–**73)	0.011*	58 (43**–**79)	56 (47**–**79)	59 (43**–**70)	1

n: absolute number; *: significant *p-value*.

## 4 Discussion

This study showed that through a 12-week pilot study, it was possible to carry out a proof-of-concept for the implementation of medical consultations via specialized video calls for patients with ACL and CD in a Brazilian public referral hospital for infectious diseases. The results point to the viability and good acceptability of this resource by patients at different clinical stages of these diseases, with the need to adapt some minimum requirements for the solution to gain scale in the service (e.g.: the offer of a digital certification for signing virtual medical documents and specific terms of consent).

The intervention was effective in promoting synchronous access for patients to their healthcare teams via the WhatsApp app. This resource, despite its informality, was widely incorporated as a means of communication by healthcare teams during the Covid-19 pandemic in Brazil (44). The simplicity and low cost of this intervention make it potentially feasible to replicate it in other LMIC contexts, like some initiatives already underway by the national government for other endemic infectious diseases (45).

In the group studied, the distance to the specialist center proved to be a significant barrier to access for the participating patients, most of whom lived in other municipalities. This finding is consistent with the broader sociodemographic profile of individuals affected by ACL and CD in Brazil, whose health conditions remain closely linked to social determinants (11, 25, 28). ACL predominantly affects populations living in rural or peri-urban areas, frequently characterized by precarious housing, poor infrastructure, and restricted healthcare access. It disproportionately impacts males engaged in agricultural, extractive, or construction activities, with the highest prevalence among economically active adults (11, 25). Similarly, CD mainly affects individuals originating from historically marginalized rural regions. Most of those currently living with CD are adults or older adult individuals who acquired the infection decades ago, prior to the successful control of vectorial transmission in Brazil (11, 27, 28). This population typically presents with low educational attainment and persistent socioeconomic vulnerabilities (11, 28).

Barriers like limited healthcare infrastructure, diagnostic challenges, and geographic remoteness hinder access to timely and specialized care in rural and underprivileged regions. For this population Telemedicine can be useful in avoiding travel time and financial costs, preventing absenteeism and loss of clinical follow-up (7, 15). In addition, in the specific scenario of the city of Rio de Janeiro, a relevant barrier to access is urban violence, which often limits the mobility of patients living in high-risk areas, as access routes to the service can be obstructed due to armed conflicts (46). Remote consultations can be an alternative in these conditions.

### 4.1 SWOT analysis

**Figure 4:**
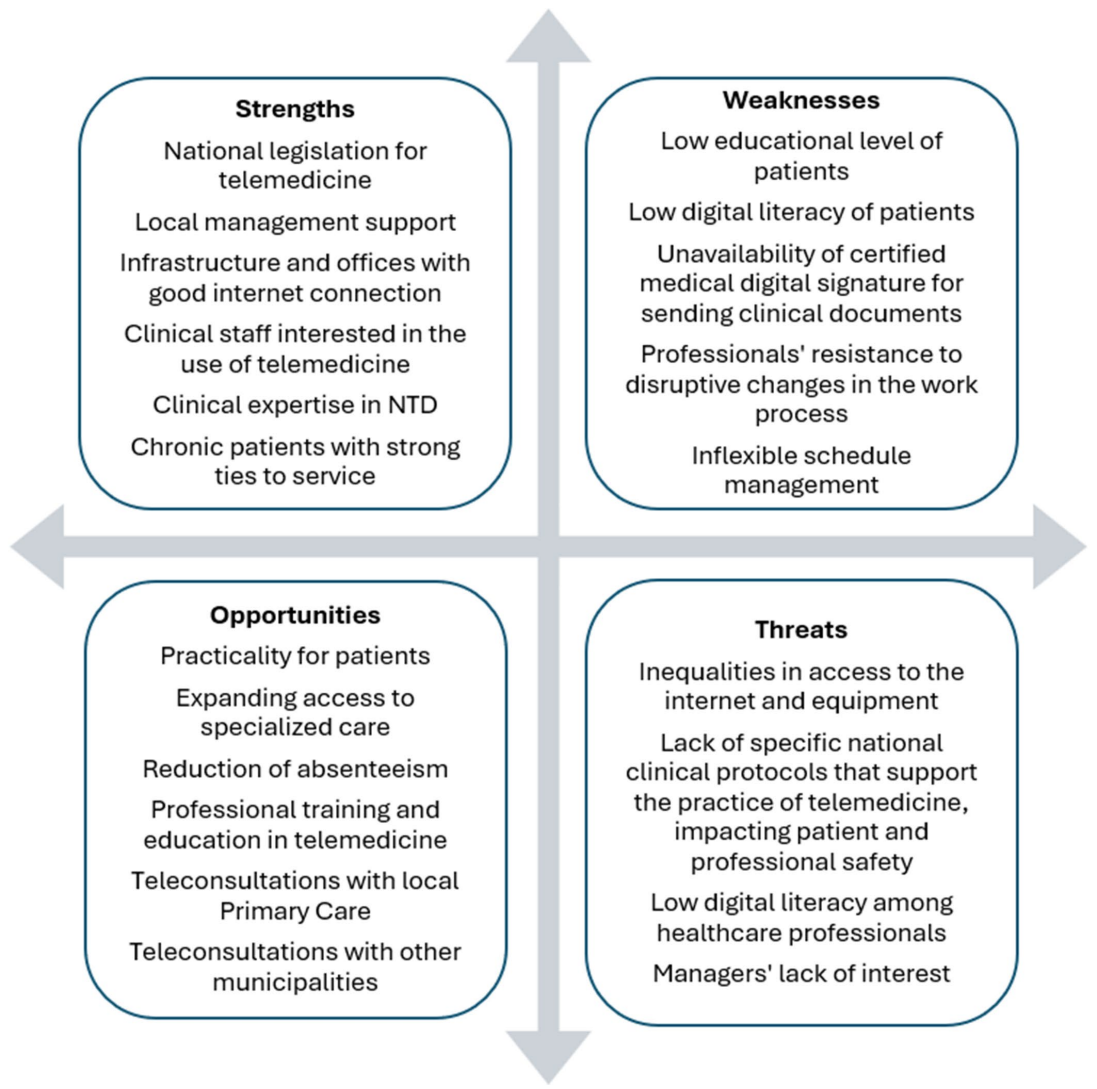
SWOT analysis matrix derived from the pilot implementation results at INI-Fiocruz.

Interest in implementation research is growing in global health, especially in LMIC, because of its potential to build practical approaches applied to real-world settings in the implementation of innovative health policies (31–33). A summary of the analysis of the results based on the SWOT technique (Strengths, Weaknesses, Opportunities, and Threats) is presented in Figure 4, signaling lessons learned and recommendations for future interventions (47).

#### 4.1.1 Strengths

Among the local contextual success factors not yet presented above is the history of successful experiences in previous remote consultations at the INI. Two prominent initiatives took place previously: one with telephone calls (48), and the other with synchronous medical teleconsultations via video calls (49). Although applied to other diseases and initially restricted to research protocols, both were incorporated into the team’s work process and remain in force to this day. This history suggests a governance context favorable to the local sustainability of telemedicine solutions.

Another important factor that may have acted as a facilitator is the long-term relationship that most professionals have with the service and that patients have with their doctors. As these are chronic diseases that require prolonged longitudinal follow-up, this bond may have favored acceptance and reliability of the teleconsultation invitation on the part of the patients.

#### 4.1.2 Weaknesses

There was a statistical association between low schooling and the need for help for the patient to carry out the teleconsultation in ACL. However, this need, which is common in populations with a higher occurrence of NTDs (3), did not prevent teleconsultations from being carried out in this study. This data corroborates the recommendations that an appropriate telemedicine model for low-resource settings should consider the specific needs of users (6, 7, 33), offering simplified access steps and possibly support teams for scheduling, as well as the instructional involvement of friends, family or community health teams who can offer on-site support.

The incorporation of telemedicine can lead to disruptive changes in the care routine, altering scheduling dynamics, duration of appointments, and clinical follow-up protocols, as well as generating insecurity for professionals and patients who are already used to the routine (6, 7, 33). In the context of the INI-Fiocruz, since it is a research center, the need to collect samples in its laboratory or carry out tests locally emerged as factors of resistance on the part of the clinical staff in the exploratory stage of the study. In this sense, strategic alignment with the teams is recommended to guarantee agenda management that is coherent with care needs, without disrupting the work process.

Limitations for evaluating clinical parameters in ACL and CD by telemedicine also need to be evaluated according to the patient’s profile and the reliability of self-measurement. For example, evaluating body weight in the progression of edema of cardiac origin in CD requires a calibrated scale at the patient’s home, as well as instruments for measuring blood pressure. The investigation of mucosal lesions in the ACL necessarily requires an in-person visit for oroscopy, rhinoscopy, or laryngoscopy (25). Specific protocols for virtual care are recommended to align the criteria for choosing the type of care, in a manner consistent with internationally recommended good clinical practices.

#### 4.1.3 Opportunities

##### 4.1.3.1 Opportunities for using telemedicine in ACL clinical care

The literature demonstrates that teledermatology is an efficient tool in the care of infectious skin diseases, offering a rapid and effective response, including for ACL (15, 50, 51). Previous studies have described the feasibility of implementing asynchronous mobile health tools for diagnosis and follow-up of ACL in the Americas region, as presented by Cossio and colleagues in rural areas of Colombia (52).

In this study, there was excellent adherence by health professionals and patients with ACL to teleconsultations (95.6%, 22/23). It is worth noting that telemedicine has demonstrated particular usefulness in two specific situations: 1. at the beginning of treatment, when doubts are more frequent, offering the patient greater availability to contact the doctor at shorter intervals than the usual follow-up; 2. to monitor the healing process more closely and ensure more consistent clinical follow-up of the lesion, optimizing the detection of a possible therapeutic failure or complication in refractory cases of patients living in distant areas.

##### 4.1.3.2 Opportunities for using telemedicine in CD clinical care

Recent studies have shown that telemedicine interventions can significantly reduce mortality and hospitalization rates in patients with heart disease (53, 54). In our study, there was lower adherence of patients with severe CD to teleconsultation, which can be explained by the reduced availability of the professional’s schedule, which compromised the care of this group. Another specific aspect is that the service has a respiratory physiotherapy center for severe CDs, which already requires these patients to travel there frequently, making them less interested in remote consultations.

However, it is worth noting that the use of telemedicine could be useful in these patients, especially in the following situations: 1. monitoring patients with compensated heart failure for early detection of decompensations, sending medical orders and updated prescriptions without the need for travel; 2. patients who use anticoagulant medications for serious arrhythmias resulting from CD need to periodically send requests for laboratory tests on peripheral blood, which can be managed by the team remotely, directing the collection to public laboratories close to the patient’s residence.

For patients with Indeterminate CD, the benefits of remote clinical monitoring involved: 1. practicality, as it can be carried out even in the workplace or during a trip (two situations that occurred with patients in this group); 2. the possibility of shortening the follow-up interval to check test results or clinical evolution; 3. the potential improvement in patient retention rates in the service for appropriate regular monitoring of the risks of CD progression.

##### 4.1.3.3 Future opportunities regarding care networks

Telemedicine integration between primary health care units and specialized centers can be a highly useful resource for qualifying care and optimizing the patient journey in SUS care networks (14, 18, 44). The use of teleconsultations between the studied hospital and the local primary health care or other specialized services was not evaluated in this pilot. But this resource represents a relevant opportunity given the lower knowledge of general practitioners about ACL and CD, even in endemic areas. Teleconsultation support for the initial evaluation of a suspected diagnosis may have an impact on reducing the interval between treatment initiation and on better outcomes at an individual and community levels (15, 51).

In addition, patients from other municipalities could benefit from teleconsultations with the reference specialist at the care coordination center. This relationship could be beneficial in bringing the levels of care closer together and strengthen the continuity of care for these diseases at a regional and inter-municipal level of the SUS (16, 18, 25, 28).

Training of human resources in telemedicine is another opportunity to be explored that represents a challenge in LMIC, being an aspect of global recommendation to produce long-term advances for digital health in health systems (6, 7, 30, 33). The use of teleconsultations by local postgraduate programs can help train young doctors and multi-professional health teams in remote care competencies and skills for patients with infectious diseases, as well as open up space for new lines of in-service research.

#### 4.1.4 Threats

In Brazil, there is greater detection and mortality from NTDs in smaller municipalities (11), where there are often greater limitations to accessing health care. Telemedicine incorporation is a challenge in these regions due to structural difficulties such as the availability of electronic devices and the stability of the internet connection (3, 7, 30).

Therefore, telemedicine cannot be the only care offered to patients in low-income and low-connectivity regions, and there must be local protocols that indicate the clinical limitations for offering this modality. For example, in ACL or CD cases, monitoring the side effects of a certain treatment or checking the laboratory results of an asymptomatic patient could be done remotely, while the appearance of new symptoms may require in-person evaluation.

The use of digital devices is an alternative when it is not possible to travel for an in-person physical examination, but an irresponsible replacement of care modalities could compromise patient safety and increase inequities and should be a concern for managers and decision-makers. In addition, privacy measures associated with teleconsultations must be rigorously implemented to protect patient data and maintain trust in digital health services. Evidence-based regulations and good practice guidelines developed in conjunction with specialty societies emerge as an important research agenda for the current moment of expansion of digital health in the Brazilian SUS (18).

### 4.2 Limitations

This pilot study was conducted with a relatively small and specific population, which does not capture the variability inherent across diverse epidemiological or healthcare scenarios. The limited representativeness of the population included in this study constrains the general application of the findings, which are also specific to CD and ACL and are not intended to be extrapolated to other NTDs or healthcare settings. Nevertheless, the synthesis of these findings may provide valuable insights for the design and implementation of future interventions in analogous settings.

The study did not collect data on the family income, race, or ethnicity of participants, which weakens socioeconomic analyses. However, education data can provide an estimate of the social pattern studied.

Another limitation to the analysis of the acceptability of telemedicine was the lack of evaluation of the satisfaction of health professionals after the intervention. Likewise, the evaluation of accuracy or clinical resolution was not the objective of this study, and these aspects should be considered in new studies with appropriate designs.

## 5 Conclusion

The intervention developed in this pilot implementation study proved to be both feasible and effective in facilitating synchronous access for patients to specialized healthcare teams. The findings indicate high acceptability of telemedicine for the management of ACL and CD in the context studied, with potential for the solution to be scaled across the service.

Future research to compare remote and in-person consultations regarding diagnostic accuracy and clinical outcomes for ACL and CD is necessary to ensure patient safety in choosing the best care modality according to the clinical presentation. Conducting cost-effectiveness analysis of telemedicine as a healthcare strategy is also essential for informing evidence-based policymaking and ensuring the long-term sustainability and effective integration of these within health systems.

## Data Availability

The original contributions presented in the study are included in the article/Supplementary material, further inquiries can be directed to the corresponding authors.
