# Supplementary material for: Telemedicine in the clinical care of Chagas disease and American cutaneous leishmaniasis: pilot study in a public referral hospital in Brazil
Source: Front Public Health. 2025 Jun 26;13:1616368. doi: 10.3389/fpubh.2025.1616368 (PMC12243272; doi:10.3389/fpubh.2025.1616368)
Supplement: Supplementary file 1 [file Data_Sheet_1.pdf]

# Informed Consent Form for Patients

Por favor, complete o inquérito abaixo.

Obrigado!

TERMO DE CONSENTIMENTO LIVRE E ESCLARECIDO PARA PARTICIPAÇÃO NA PESQUISA - PACIENTES AUTORIZAÇÃO PARA COLETA DE DADOS DOS QUESTIONÁRIOS E REALIZAÇÃO DA CONSULTA MÉDICA POR TELEMEDICINA

INSTITUIÇÃO: INSTITUTO DE PESQUISA CLÍNICA EVANDRO CHAGAS - FIOCRUZ. COORDENADORA DA PESQUISA: CLÁUDIA MARIA VALETE ROSALINO, ENDEREÇO: Av. Brasil 4365 - Manguinhos - Rio de Janeiro - RJ - CEP 21040-900, TELEFONES +55 (021) 3865-9525/9609 /9541

PESQUISA: Telemedicina no cuidado a Doenças Tropicais Negligenciadas na Comunidade de Países de Língua Portuguesa - proposta para implementação de um protocolo assistencial de referência

PROPÓSITO DO ESTUDO Prezado participante, este estudo tem como objetivo principal desenvolver um modelo de protocolo de utilização da Telemedicina em língua portuguesa para apoiar um serviço de cuidados a Doenças Tropicais Negligenciadas (DTN), que poderá servir de referência para adaptação em outros contextos da Comunidade de Países de Língua Portuguesa (CPLP), sob a perspectiva da cooperação global em Saúde Digital.
A primeira etapa do estudo já foi finalizada e caracterizou dois serviços públicos de referência em cuidados a DTN em dois países (Angola e Brasil) quanto à conjuntura para utilização de recursos de Telemedicina, investigando suas principais barreiras e potenciais de aplicação nestes diferentes cenários de prática. A atual etapa do estudo investiga a viabilidade de se aplicar a Telemedicina no cuidado a duas DTNs atendidas nos ambulatórios do Instituto Nacional de Infectologia Evandro Chagas (INI/Fiocruz): a Doença de Chagas e a Leishmaniose Tegumentar Americana, a partir de um estudo piloto.

CONVITE PARA O ESTUDO Pelo presente documento você está sendo convidado/a para participar desta pesquisa por ser usuário/paciente do INI/Fiocruz que recebe assistência clínica direta ou indireta a uma destas doenças. Para participar, você precisa: Ter diagnóstico confirmado de doença de Chagas ou leishmaniose cutânea, ter mais de 17 anos; possuir um dispositivo fixo ou móvel com acesso à internet e WhatsApp instalado em casa ou no trabalho; não apresentar nenhuma condição de saúde para a qual o médico responsável tenha preferido o acompanhamento presencial, e aceitar este termo de consentimento livre e esclarecido, demonstrando interesse em realizar o acompanhamento com o médico responsável via telemedicina nas condições explicitadas. Sua permissão para participar da pesquisa será concedida ao clicar no botão abaixo que diz "CONCORDO EM PARTICIPAR DESTA PESQUISA".

Caso você decida participar, a sua participação consistirá em: 1. responder a um questionário agora (avaliação pré-consulta), de forma presencial, pelo tablet da pesquisadora; 2. responder a um questionário após a realização da teleconsulta (avaliação pós-consulta), de forma remota - cada questionário leva em média 3 minutos para ser respondido; e. 3. autorizar a realização de uma Teleconsulta médica pela plataforma do aplicativo Whatsapp com a sua equipe de referência do serviço INI/Fiocruz.

A teleconsulta não será gravada. O link para acessar a teleconsulta e o link para acessar o questionário pós-consulta serão enviados remotamente para seu contato de telefone por whatsapp ou mensagem. Estes dados de contato serão fornecidos por você no questionário estritamente para a finalidade desta pesquisa. As informações que serão armazenadas nesta teleconsulta são as mesmas de uma consulta presencial, ou seja, serão registradas no prontuário eletrônico institucional do INI-Fiocruz com garantia de privacidade e confidencialidade através do Código de Ética Médica e do código de ética em pesquisa. Quanto às informações obtidas nos questionários, a pesquisadora se responsabilizará pelo armazenamento adequado dos dados coletados, bem como pelos procedimentos para assegurar o sigilo e a confidencialidade das informações do participante da pesquisa. Serão guardadas em local seguro pelos investigadores, no prazo previsto pelas normas vigentes, respeitando a confidencialidade da informação obtida. Serão armazenados em arquivos digitais e somente terão acesso às mesmas a pesquisadora e seus orientadores. Ao final da pesquisa, todo material será mantido em arquivo, por pelo menos 5 (cinco) anos, conforme Resolução 466/12 do Conselho Nacional de Saúde e orientações do CEP/INI.

PARTICIPAÇÃO VOLUNTÁRIA E LIBERDADE PARA DESISTIR A decisão de participar ou não deste estudo é voluntária, isto é, ela não é obrigatória, e você tem plena autonomia para decidir se quer ou não participar, bem como retirar sua participação a qualquer momento. Você não será penalizado de nenhuma maneira caso decida não consentir sua participação. A qualquer momento você poderá solicitar da pesquisadora mais informações sobre sua participação e/ou sobre a pesquisa, o que poderá ser feito através dos meios de contato explicitados neste Termo.

RISCOS Sua participação não envolve riscos físicos. Os riscos e/ou desconfortos relacionados à sua participação neste estudo estão relacionados a possíveis constrangimentos ao responder alguma pergunta ou quebra do sigilo/anonimato dos dados. Uma vez concluída a coleta de dados, eles serão descarregados e salvos em um dispositivo eletrônico local, apagando todo e qualquer registro de qualquer plataforma virtual, ambiente compartilhado ou "nuvem", minimizando este possível risco. O acesso aos resultados é limitado apenas aos pesquisadores, que se comprometem com a garantia do sigilo, do anonimato e da segurança dos dados obtidos e do material. O senhor(a) tem o direito de buscar indenização diante de eventuais danos.

BENEFÍCIOS Você terá como benefício a oportunidade de ter o atendimento médico online além das consultas presenciais necessárias ao seu acompanhamento, remotamente (a distância) e sem a necessidade de deslocamento do seu domicílio. Além disso, a partir dessa pesquisa, a equipe poderá desenvolver inovações tecnológicas para prestação de assistência remota à pacientes com DTN no INI-Fiocruz.

CONFIDENCIALIDADE Serão garantidas a(o) senhor(a) a confidencialidade e a privacidade das informações por você prestadas. Os resultados da pesquisa divulgados não apresentarão seus dados de forma direta nem indireta. Todas as informações recolhidas serão tratadas de forma confidencial e anônima: a sua identificação nunca será revelada em nenhum relatório ou publicação, e seus médicos e profissionais de saúde não terão acesso aos dados identificados de sua avaliação.

COMPENSAÇÃO A participação nesta pesquisa não implica custos para você e não haverá nenhum tipo de pagamento ou reembolso de sua parte.

Em caso de dúvida quanto à condução ética do estudo, entre em contato com o Comitê de Ética em Pesquisa INI/Fiocruz (CEP INI/Fiocruz). Esta é a instância que tem por objetivo defender os interesses dos participantes da pesquisa em sua integridade e dignidade dentro de padrões éticos. Dessa forma o comitê tem o papel de avaliar e monitorar o andamento do projeto de modo que a pesquisa respeite os princípios éticos de proteção aos direitos humanos, da dignidade, da autonomia, da não maleficência, da confidencialidade e da privacidade.

Comitê de Ética em Pesquisa - CEP INI/Fiocruz:
Endereço: Av. Brasil, 4365 Manguinhos - 21040-360 Rio de Janeiro - RJ
Telefone: +55 (21) 3865-9585 E-Mail: cep@ini.fiocruz.br
Se desejar, consulte ainda a Comissão Nacional de Ética em Pesquisa (Conep):
Tel: +55 (61) 3315-5878 / 3315-5879
E-Mail: conep@saude.gov.br

Contato com a pesquisadora coordenadora responsável
Nome: Cláudia Maria Valete
Endereço: Av. Brasil, 4365, Manguinhos - Rio de Janeiro/RJ - 21040-360
Telefone: +55 (21) 3865-9525 / 3865-9609 / E-mail: claudia.valete@ini.fiocruz.br

DECLARAÇÃO DE CONSENTIMENTO LIVRE E ESCLARECIDO
Após a leitura do presente termo, caso o aceite, selecione a opção "CONCORDO EM PARTICIPAR DESTA PESQUISA" localizada abaixo. Ao fazer isso você informa que leu e está de acordo com o apresentado, e espera-se que você responda os questionários até o final. Sua teleconsulta será agendada e o link de acesso será enviado aos endereços de e-mail e/ou telefone fornecidos por você. Caso não aceite participar desta pesquisa, basta comunicar à pesquisadora e fechar esta tela.

Ao clicar no botão abaixo, o(a) senhor(a) concorda em participar da pesquisa de acordo com as informações registadas neste Termo. A qualquer momento você poderá desistir de participar da pesquisa sem qualquer prejuízo pessoal, seu desejo será respeitado e você não será penalizado.
Você receberá uma cópia impressa deste Termo de Consentimento assinado pela pesquisadora.

{INFORMED CONSENT FORM FOR RESEARCH PARTICIPATION - PATIENTS
AUTHORIZATION FOR DATA COLLECTION FROM QUESTIONNAIRES AND MEDICAL CONSULTATION VIA TELEMEDICINE

INSTITUTION: Instituto de Pesquisa Clínica Evandro Chagas - FIOCRUZ
PRINCIPAL INVESTIGATOR: Cláudia Maria Valete Rosalino
Address: Av. Brasil, 4365 - Manguinhos - Rio de Janeiro - RJ - ZIP Code 21040-900
Phone Numbers: +55 (21) 3865-9525 / 9609 / 9541

RESEARCH TITLE: Telemedicine in the Care of Neglected Tropical Diseases in the Community of Portuguese Language Countries (CPLP) - A Proposal for the Implementation of a Reference Care Protocol

PURPOSE OF THE STUDY
Dear participant, the primary objective of this study is to develop a telemedicine protocol in Portuguese to support healthcare services for Neglected Tropical Diseases (NTDs). This protocol may serve as a reference for adaptation in other contexts within the Community of Portuguese Language Countries (CPLP), within the framework of global cooperation in Digital Health. The first phase of this study has been completed and consisted of the characterization of two public healthcare services specializing in NTDs in two countries (Angola and Brazil), analyzing the feasibility, barriers, and potential applications of telemedicine in these different operational contexts. The current phase evaluates the feasibility of applying telemedicine in the care of two NTDs managed at the outpatient clinics of the Instituto Nacional de Infectologia Evandro Chagas (INI/Fiocruz): Chagas Disease and American Cutaneous Leishmaniasis, through a pilot study.

INVITATION TO PARTICIPATE

You are being invited to participate in this research because you are a patient at INI/Fiocruz receiving care for one of these conditions. To be eligible, you must: Have a confirmed diagnosis of Chagas disease or cutaneous leishmaniasis; Be 18 years of age or older; Have access to an internet-connected device (computer or smartphone) with WhatsApp installed at home or at work; Not present any health condition that, in the physician's assessment, requires in-person follow-up; Agree to this Informed Consent Form, thereby expressing your willingness to participate in the medical consultation via telemedicine under the conditions described.

Your authorization to participate will be granted by clicking the button below that states "I AGREE TO PARTICIPATE IN THIS RESEARCH." If you agree to participate, your involvement will consist of: 1. Completing a pre-consultation questionnaire (in person, via the researcher's tablet); 2. Completing a post-consultation questionnaire (remotely); each questionnaire will take approximately 3 minutes;3. Authorizing the medical consultation to be conducted via telemedicine using the WhatsApp platform, with your regular medical team from INI/Fiocruz.

The teleconsultation will not be recorded. The link for the teleconsultation and the post-consultation questionnaire will be sent to your phone via WhatsApp or text message. This contact information will be collected solely for the purpose of this study.

Information recorded during the teleconsultation will be the same as that of a face-to-face consultation, documented in your official electronic medical record at INI-Fiocruz, following privacy and confidentiality standards, as per the Medical Ethics Code and research ethics regulations. Data collected from questionnaires will be securely stored by the research team, with procedures in place to ensure confidentiality. Data will be kept in secure digital files, accessible only to the principal investigator and her supervisors, and stored for at least five (5) years as required by Brazilian National Health Council Resolution No. 466/2012 and the INI Research Ethics Committee (CEP/INI) guidelines.

VOLUNTARY PARTICIPATION AND RIGHT TO WITHDRAW
Your participation is entirely voluntary. You are free to decide whether or not to participate and may withdraw your consent at any time without any penalty or loss of benefits to which you are otherwise entitled. You may also request further information about your participation or the study from the research team at any time using the contact details provided in this document.

RISKS
There are no physical risks associated with participating in this research. Potential risks are minimal and relate primarily to possible discomfort in answering questions or breaches of confidentiality. To mitigate this, all data will be downloaded to a secure local device immediately after collection and deleted from any cloud or shared platforms. Access to the data will be strictly limited to the research team, ensuring anonymity, privacy, and data protection. You retain the right to seek compensation should any harm occur.

BENEFITS
Participants may benefit from the convenience of receiving medical consultations remotely, in addition to regular in-person appointments, thereby reducing the need for travel. Furthermore, this study contributes to the development of technological innovations aimed at improving remote care for patients with NTDs at INI-Fiocruz.

CONFIDENTIALITY
Your confidentiality and privacy will be rigorously protected. The results of this study will be presented in aggregate form, and no information will be disclosed that could directly or indirectly identify you. All information will be treated anonymously and confidentially. Your identification will never appear in any report, presentation, or publication resulting from this research. Clinical staff outside the research team will not have access to your identified data.

COMPENSATION
Participation in this study does not entail any financial costs, nor will participants receive any form of payment or reimbursement.

ETHICAL REVIEW AND CONTACT INFORMATION
If you have any concerns regarding the ethical aspects of this study, you may contact the INI-Fiocruz Research Ethics Committee (CEP/INI), which is responsible for ensuring that this research respects ethical principles, including the protection of human rights, dignity, autonomy, non-maleficence, confidentiality, and privacy.

Research Ethics Committee - CEP INI/Fiocruz
Address: Av. Brasil, 4365, Manguinhos - 21040-360, Rio de Janeiro - RJ - Brazil
Phone: +55 (21) 3865-9585
Email: cep@ini.fiocruz.br
You may also contact the National Research Ethics Commission (CONEP):
Phone: +55 (61) 3315-5878 / 3315-5879
Email: conep@saude.gov.br

CONTACT WITH THE PRINCIPAL INVESTIGATOR
Name: Cláudia Maria Valete Rosalino
Address: Av. Brasil, 4365, Manguinhos - Rio de Janeiro - RJ - 21040-360 - Brazil
Phone: +55 (21) 3865-9525 / 9609

Email: claudia.valete@ini.fiocruz.br

DECLARATION OF INFORMED CONSENT
After reading this document, if you agree to participate, please click "I AGREE TO PARTICIPATE IN THIS RESEARCH" below. By doing so, you indicate that you have read, understood, and agree with the terms presented. You are expected to complete the questionnaires and will receive the link for the teleconsultation at the contact details you provide. If you do not wish to participate, simply inform the researcher and close this screen. By clicking the button below, you confirm your voluntary agreement to participate under the terms described. You may withdraw from the study at any time without any personal consequences or penalties. You will receive a printed copy of this Informed Consent Form, signed by the researcher.}

---

1) ◯ CONCORDO EM PARTICIPAR DESTA PESQUISA. Declaro q[illegible] estou ciente e compreendo as informações apresentadas neste Termo de Consentimento Livre e Esclarecido. Autorizo o uso do WhatsApp para comunicação com os profissionais de saúde vinculados a este projeto de pesquisa, exclusivamente para atividades relacionadas ao seu âmbito, incluindo agendamento e realização de teleconsultas, troca de informações clínicas e envio de documentos médicos. Estou ciente de que poderão ser tratados dados pessoais sensíveis, incluindo informações de saúde, em conformidade com a Lei nº 13.709/2018 (Lei Geral de Proteção de Dados - LGPD), para finalidades assistenciais e de pesquisa acadêmica, conforme disposto na Resolução CNS nº 466/2012 e na Lei nº 14.510/2022 (Lei da Telessaúde). Estou ciente das limitações da modalidade de atendimento remoto, e de que poderá ser necessário uma nova consulta presencial para complementar minha avaliação clínica. { I AGREE TO PARTICIPATE IN THIS RESEARCH. I hereby declare that I am aware of and understand the information presented in this Informed Consent Form. I authorize the use of WhatsApp for communication with the healthcare professionals involved in this research project, exclusively for activities related to its scope, including the scheduling and execution of teleconsultations, exchange of clinical information, and the transmission of medical documents. I am aware that sensitive personal data, including health-related information, may be processed in accordance with Brazilian Law No. 13,709/2018 (General Data Protection Law - LGPD) for purposes related to healthcare and academic research, in compliance with Brazilian National Health Council Resolution No. 466/2012 and Law No. 14,510/2022 (Telehealth Law). I acknowledge the limitations inherent to remote medical consultations and understand that an additional in-person consultation may be necessary to complement my clinical evaluation.}
